# Interactions between classic psychedelics and serotonergic antidepressants: Effects on the acute psychedelic subjective experience, well-being and depressive symptoms from a prospective survey study

**DOI:** 10.1177/02698811231224217

**Published:** 2024-01-27

**Authors:** Jessica Barbut Siva, Tommaso Barba, Hannes Kettner, Joanna Kuc, David J Nutt, Robin Carhart-Harris, David Erritzoe

**Affiliations:** 1 Centre for Psychedelic Research, Department of Medicine, Imperial College London, London, UK; 2 Experimental Psychology, University College London, London, UK; 3 Psychedelics Division – Neuroscape, Department of Neurology, University of California San Francisco, San Francisco, CA, USA

**Keywords:** Serotonergic antidepressants, classic psychedelics, subjective experience, well-being, depressive symptoms

## Abstract

**Background:**

There is growing evidence for the therapeutic effects of psychedelics. However, it is still uncertain how these drugs interact with serotonergic antidepressants (serotonin reuptake inhibitors (SRIs)).

**Objective:**

This study explores the interaction between psychedelics and SRIs in terms of therapeutic effects. The objective is to compare acute psychedelic effects and subsequent changes in well-being and depressive symptoms among ‘SRI −’ individuals (not on psychiatric medication) and ‘SRI +’ individuals (undergoing SRI treatment).

**Methods:**

Using prospective survey data, the study employs multivariate analysis of covariance (MANCOVA) and linear mixed effect models to analyse subjective differences and changes in well-being and depressive symptoms pre- and post-psychedelic experiences.

**Results:**

Results indicate that ‘SRI −’ participants experience significantly more intense subjective effects compared to ‘SRI +’ participants (*F* = 3.200, *p* = 0.016) in MANCOVA analysis. Further analysis reveals ‘SRI –’ individuals report stronger mystical (18.2% higher, *p* = 0.048), challenging (50.9% higher, *p* = 0.001) and emotional breakthrough experiences (31.9% higher, *p* = 0.02) than ‘SRI +’ individuals. No differences are observed in drug-induced visual effects (*p* = 0.19). Both groups exhibited similar improvements in well-being and depressive symptoms after the psychedelic experience.

**Conclusion:**

Individuals presumed to be on serotonergic antidepressants during psychedelic use display reduced subjective effects but similar antidepressant effects compared to those not undergoing SRI treatment. Further controlled research is needed to comprehend the interplay between serotonergic antidepressants and psychedelics, illuminating potential therapeutic benefits and limitations in clinical contexts.

## Introduction

There is a resurgence of research into the use of classic psychedelic compounds such as psilocybin, lysergic acid diethylamide (LSD), *N,N*-dimethyltryptamine (DMT) and mescaline within clinical settings. Growing evidence suggests that psychedelics administered in controlled settings can produce rapid and sustained symptom improvements in depression (Carhart-Harris et al., 2016, 2021; Davis et al., 2021), obsessive-compulsive disorder (OCD) (Moreno et al., 2006) and anxiety disorders (Griffiths et al., 2016; Holze et al., 2023).

In current clinical research involving psychedelic drugs, many subjects are psychiatric patients who may have been using selective serotonin reuptake inhibitor (SSRI) and serotonin and noradrenaline reuptake inhibitor (SNRI) medications (e.g. to treat symptoms of depression, anxiety, OCD or post-traumatic stress disorder (PTSD)) for extended periods of time prior to the clinical study, due to their widespread prescription in psychiatric populations (Luo et al., 2020). SSRIs and SNRIs, which are classes of drugs that belong to the serotonin reuptake inhibitors (SRIs) category, exert their effects by blocking serotonin (5-HT) reuptake (Stahl, 2013). This blockade is thought to enhance the serotonin 1A (5-HT_1A_) receptor signalling pathway, which fosters stress tolerance and resilience (Carhart-Harris and Nutt, 2017).

Given the lack of established safety data for combined psilocybin and SRIs, patients are typically required to stop taking SRIs for at least 2 weeks before the start of the trial (Carhart-Harris et al., 2021; Davis et al., 2021; Malcolm and Thomas, 2022). This precaution is taken in part due to both a lack of fully established evidence for safety and to previous case reports that suggest chronic use of SRIs can reduce the therapeutically important subjective effects of psychedelics (Bonson et al., 1996; Strassman, 1992). More precisely, an early study published two case reports on the interaction between SRIs and psychedelics (Strassman, 1992). Both patients reported a diminished subjective sensitivity to either psilocybin or LSD. Another early observational study investigated the possible interaction between chronically used SRIs and LSD in subjects who volunteered to be interviewed via standardised questionnaires (Bonson et al., 1996). It was found that 88% of the patients reported a decreased LSD experience or a virtual elimination of their response to LSD after using SRIs for over 3 weeks, displaying congruent results to the ones reported by Strassman (1992).

A recent randomised controlled trial further investigated the potential pharmacological interactions between psychedelics and SRIs by assessing the acute effects of psilocybin in healthy volunteers who had undergone 14 days of pre-treatment with escitalopram (SSRI) or placebo (Becker et al., 2022). Results demonstrated that pre-treatment with escitalopram reduced the physiological effects of psilocybin and bad drug consequences such as anxiety and cardiovascular effects. However, it had no consequences on the positive effects induced by psilocybin. This study suggested that SRIs and psilocybin could be safely and effectively administered together, although the short duration of escitalopram treatment and the population consisting of healthy subjects limited the generalisability of the results. Nevertheless, a recent retrospective observational study assessing the potential interaction between SRIs and psilocybin found contradictory results, showing that concurrent use of SRIs weakened psilocybin’s subjective effects in about half of the study subjects (Gukasyan et al., 2023)

Although there is extensive research on the interactions between antidepressants and other prescribed medications (Low et al., 2018; Nieuwstraten et al., 2006), to our knowledge only two modern studies investigated the interaction between SRIs and psychedelics, presenting partially contradictory results. To enrich the evidence base for this important topic, the present study, based on prospective survey data collected from people consuming psychedelics in naturalistic settings, aims to explore (1) potential differences in acute psychedelic subjective effects between individuals with a self-reported psychiatric diagnosis currently being treated with SRIs and those who have never used such medications and (2) potential difference in before–after changes in self-rated depressive symptoms and well-being after naturalistic use of psychedelic drugs between these two populations. The results of this study could have implications for modifying research design and inclusion criteria for certain clinical studies and for informing future medical use to maximise treatment efficacy and positive outcomes.

## Methods

The present study combines data sets from three different survey samples from the Centre for Psychedelic Research’s web survey portfolio. The first data set (Cohort 1) was obtained from a large prospective cohort study (Haijen et al., 2018), where a software platform was used to collect large amounts of data. This platform was created to enable volunteers to complete a number of questionnaires if they were planning to take psychedelics in the near future. Depending on the subjects’ expected psychedelic experience date, surveys were sent automatically to them at a specific time interval. The second data set (Cohort 2) was a modified version of the initial Cohort 1 study with some additional adjustments (Haijen et al., 2018) – the data were collected in the same manner as for Cohort 1. The third data set (Ceremony study) was obtained from a study investigating the effects of psychedelics taken in ceremonial or group retreat settings (Kettner et al., 2021). Subjects for these surveys were recruited from various media platforms, and for Cohort 3 also via study advertisements by the involved retreat centres. The online survey platform Alchemer was used to collect data from subjects at different time points. The web-based data collection approach that has been used for all studies provided the opportunity to collect a large amount of data in an observational and naturalistic manner.

All studies were approved by the Joint Research Compliance Office and Imperial College Research Ethics Committee at Imperial College London.

### Subjects

The above-listed studies had similar inclusion criteria. Survey subjects needed to be at least 18 years old, have good comprehension of the English language and plan on taking serotonergic classic psychedelic drugs such as psilocybin/magic mushrooms/truffles, LSD/1-propionyl-lysergic acid diethylamide (1P-LSD), DMT, 5-methoxy-*N,N*-dimethyltryptamine (5-MeO-DMT), ayahuasca, mescaline, 2,5-dimethoxy-4-bromophenethylamine (2C-B) or other drugs that have a similar mechanism of action, and plan to consume psychedelics in naturalistic settings or in participating in a psychedelic retreat or ceremony. For the present study, only subjects who self-reported to have at least one psychiatric condition and who used classic psychedelics during their experience were included in the analysis. Subjects who reported at least one psychiatric condition were then divided into two groups: (1) subjects who were never treated with a psychiatric medication (defined as ‘SRI –’) and (2) subjects who reported to be currently treated with SRIs (defined as ‘SRI +’). Details regarding the participant allocation to the two groups in the current study (‘SRI –’ and ‘SRI +’) are provided in Section ‘Psychiatric history and medication’.

### Study design and timepoints

**Figure 1.**
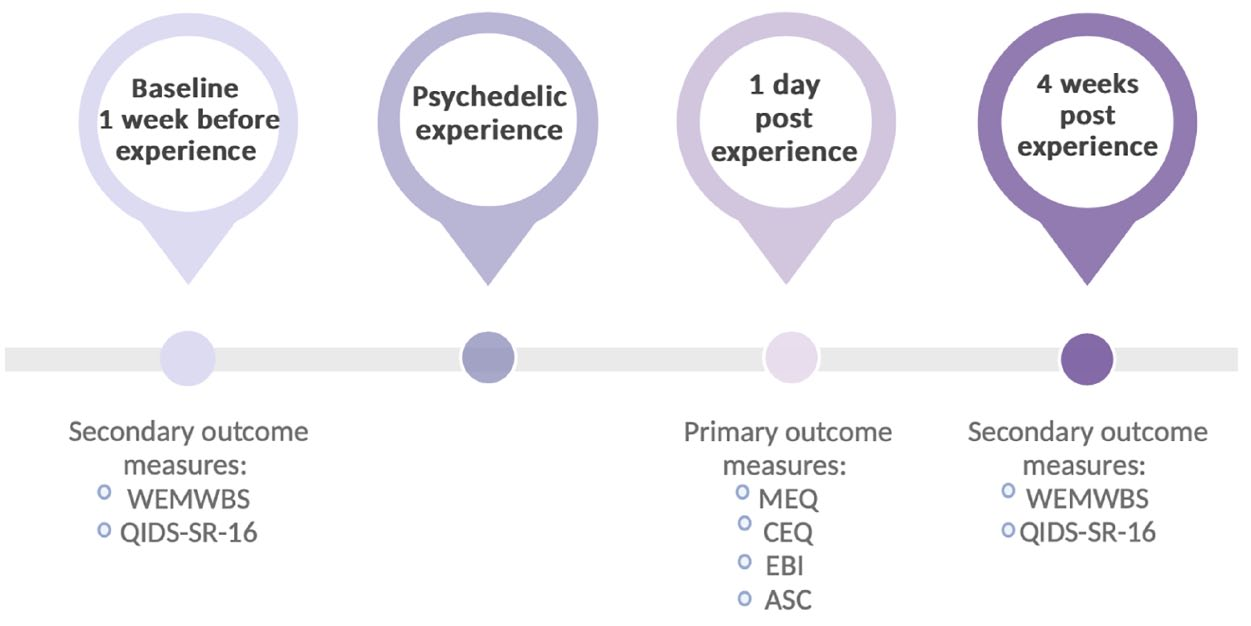
Study timeline. The first set of surveys was filled out at baseline time points which took place 1 week before the psychedelic experience. As the secondary outcome measure of the study, participants also filled out the Warwick-Edinburgh Mental Well-being Scale (WEMWBS) and 16-item Quick Inventory of Depressive Symptomatology Self-Report (QIDS-SR-16) questionnaires. Subjective experience questionnaires were sent out to survey participants 1 day after the psychedelic experience where they had to fill out Mystical Experience Questionnaire (MEQ), Challenging Experience Questionnaire (CEQ), Emotional Breakthrough Inventory (EBI) and Visual subscales of the Altered States of Consciousness Questionnaire (ASC-Vis), questionnaires which served the primary outcome measures of the study. Lastly, to assess the well-being and depressive symptoms changes, participants filled out the same WEMWBS and QIDS-SR-16 questionnaires again 4 weeks after their psychedelic experience.

The prospective cohort studies (Haijen et al., 2018) and the retreat ceremony study (Kettner et al., 2021) had a design with five different time points for data collection. However, since the present study had a different focus, only three timepoints were included in the design (Figure 1). The first timepoint was the baseline which was collected 1 week before the psychedelic experience date. At this timepoint, demographic data such as gender, age, education level, employment status, psychiatric condition, history of drug use and previous SRI use were collected. Subjects also filled out questionnaires assessing well-being and depressive symptoms. The second timepoint was the post-experience time point which took place 1 day after the participant’s psychedelic experience. The type of drug used (psychedelics or other drugs, and the dose) and questionnaires investigating the quality of the acute psychedelic experience were collected at this time point. The last time point was at 4-week post-experience where questionnaires measuring depressive symptoms and well-being were collected again to assess possible changes. Information about intentions for the psychedelic experience used as covariates in this paper was collected from 1-day pre-experience.

### Questionnaires

#### Psychiatric history and medication

During baseline, subjects were asked the following question ‘Are you currently diagnosed with one of the following psychiatric illnesses by a doctor or psychologist?’. Possible responses were ‘Major depressive disorder (MDD)’, ‘Bipolar depression’, ‘Schizophrenia’, ‘Anxiety disorder’, ‘Substance use disorder’, ‘Alcohol dependence’, ‘Hallucinogen persisting perception disorder (HPPD)’, ‘Psychotic disorder’, ‘Personality disorder’, ‘Attention deficit hyperactivity disorder (ADHD)’, ‘OCD’, ‘Eating disorder’, ‘None of the above’. Subjects were allowed to select multiple options. Only subjects who self-reported at least one psychiatric diagnosis were included in further analyses.

Subjects’ medication history was assessed with the question ‘*Have you ever been treated with any kind of psychiatric medication (e.g. antidepressant, mood stabilisers, antipsychotics, ADHD medications)*?’ If the participant reported previous medication use, a follow-up question regarding their current use (*Are you currently using these medications?*) and specific type of their medication was also inquired (*What is your currently prescribed medication?*). If subjects answered that they were using an antidepressant, a specific question on the specific type of antidepressant was asked (*What is your currently prescribed antidepressant?*). Subjects answering no to the first question were included in the ‘SRI –’ sample, and subjects answering yes to the first and second questions and subsequently indicating they were using SRIs were included in the ‘SRI +’ sample. Due to the lack of subjects reporting to use of antidepressants other than SRIs, it was not possible to include other categories in the present study.

#### Psychological and psychopathology measures

The WEMWBS was used to assess changes in psychological well-being from baseline to the key endpoint, 4 weeks after psychedelic use. The measure includes 14 items, including positive mental health and functioning, interpersonal relationship satisfaction and happiness (Tennant et al., 2007). A sum score was calculated by adding up each item, rated on a five-point Likert scale, for a maximum of up to 70 points. To assess depressive symptoms, the QIDS-SR-16 (Rush et al., 2003) was administered to subjects at baseline and the key endpoint. Both WEMWBS and QIDS-SR-16 were assessed at baseline and 4-week post-psychedelic experience.

#### Psychedelic drug type and dose

During the post-experience survey 24 h after dosing, subjects specified the type of psychedelic compound they had taken the previous day from the following options: psilocybin/magic mushrooms, LSD/1P-LSD, DMT, 5-MeO-DMT, ayahuasca, mescaline, iboga/ibogaine and self-specified answer. However, since the present study only focused on classic psychedelics, subjects who did not use psilocybin/magic mushrooms, LSD/1P-LSD, DMT/5-MeO-DMT or ayahuasca were excluded from further analysis. Subjects also indicated the approximate drug dose taken by picking an option from the following list: a low dose (≦50 μg of LSD), a moderate dose (≦100 μg of LSD), a high dose (≦200 μg of LSD), a very high dose (≦300 μg of LSD) or an extremely high dose (>300 μg of LSD). This approach was selected to standardise the doses by estimating them in relation to LSD equivalents and comparing them across different psychedelics, as was also done in previous studies (Kuc et al., 2022; Nour et al., 2016; Roseman et al., 2019).

#### Psychedelic use setting

Subjects were asked questions about their motives or intention to take psychedelics such as therapeutic, personal growth, escape from difficult emotions or curiosity. Framework (i.e. spiritual, religious), setting (i.e. fun, party, social) and environmental factors (listening to music, disruptions, emotionally supportive individuals, the presence of strangers and others) questions were also asked to subjects. These data were collected to analyse potential confounders.

#### Subjective experience

One-day post-psychedelic experience, different facets of the subjective psychedelic experience were assessed using the MEQ, the CEQ, the EBI and the ASC-Vis. The MEQ is a questionnaire assessing the intensity of mystical-type experiences, with 30 items rated on a six-point Likert scale (Barrett et al., 2015). The total MEQ scores were calculated by taking the average of all 30 items and multiplying by 20 to provide a value between 0 and 100. The CEQ assessed the unpleasant effects of psychedelic drugs (Barrett et al., 2016). Subjects were asked to rate each item on a six-point Likert scale (0–5) and the total CEQ scores were calculated by averaging all 26 items and then multiplying by 20 to provide a value between 0 and 100. The EBI assesses the experience of emotional release and catharsis. The total score was calculated by averaging across the six items (Roseman et al., 2019). The ASC-Vis contains nine items assessing changes in visual perception rated using a 0–100 visual analogue scale (0 = not more than usual; 100 = yes, entirely or completely (Studerus et al., 2010)). The total score was calculated by averaging all nine items.

### Statistical Analysis

For the primary analysis of medication-based differences in metrics of the acute subjective experience, subjects who completed baseline and the 1-day post-experience questionnaires were included in the analysis. For the secondary analysis, investigating differences in well-being and depressive symptom changes, subjects who completed all three timepoints were included.

#### Primary analysis: Effects of SRIs use on the acute psychedelic experience

The pooled sample was grouped into ‘*SRI* –’ and ‘*SRI +*’ groups. To identify potential confounding factors between the two groups, *t*-tests were performed between SRIs-naive and current SRIs-users with the following dependent variables: psychedelic dose, number of previous psychedelic experiences, intention, elements of setting and environmental factors. Significant variables (*p* < 0.05) between the two groups were classified as potential confounder factors. Among significant confounders, multicollinearity was controlled using linear regression with a variance inflation factor (VIF) cut off point of 5 being deemed critical (James et al., 2013), warranting the exclusion of one of the collinear variables. Multivariate analysis of covariance (MANCOVA) was conducted including MEQ, CEQ, EBI and ASC-Vis scores as dependent variables and SRI medication history as the independent variable. For the Ceremony study, where a few subjects attended more than one psychedelic experience across the span of a retreat, subjects were allowed to report MEQ, CEQ and EBI scores for every psychedelic session. Therefore, to obtain a single predicted score for these subjects, averages (across sessions) for each questionnaire were used. The assumption of homogeneity of variances and covariances was determined using Box’s test (Manly and Alberto, 2016). Pillai’s trace was chosen as the specific test statistic since it is robust against MANCOVA violations, such as multivariate normality (Olson, 1974). Partial effect sizes ( $\eta_{p}^{2}$ ) were calculated to evaluate differences between SRIs-naive and SRIs-users groups (0.02 = small effect size, 0.13 = medium effect size and 0.26 or higher = large effect size (Myors et al., 2014)). *p* < 0.05 was accepted as the cut-off point for statistical significance.

#### Secondary analyses: Changes in well-being and depression

To explore whether *SRI +* subjects differed compared to *SRI −* subjects in terms of changes in well-being and depressive symptoms from before to after the psychedelic experience, separate linear mixed-effects models were defined with QIDS-SR16 and WEMWBS as the outcomes. The models took the form of:

$$\begin{array}{l} \mathrm{Outcome}\sim \mathrm{Time}\times \mathrm{Condition}\\ \;+\left(\mathrm{confounding}\;\mathrm{variables}\right)+\left(1|\mathrm{Participant}\right) \end{array}$$

The condition indicates the two study groups, namely ‘SRI +’ and ‘*SRI* −’. The model was assessed for linearity, homoscedasticity (inspection of the residuals) and normality of residuals (inspection of the Q–Q plot).

Analyses were conducted using IBM SPSS Statistics (IBM MacBook, Version 26.0) and R Studio (www.rstudio.com/) using the packages lme4 (Bates et al., 2015), lmertest and ggplot2.

## Results

### Demographics

In total, 1463 subjects signed up for different studies. After filtering out subjects who did not report a psychiatric condition (healthy subjects), who used medications other than SRIs and who did not take classic psychedelics during their experience, 161 subjects answered either baseline and 1-day post questionnaires or baseline and 4-week post-questionnaires. Most of the subjects currently self-reporting a psychiatric disorder did not report having used any medication during their lifetime (*n* = 98). On the other side, 63 subjects reported to currently use SRIs. Psilocybin, including magic mushrooms or truffles, was the most used psychedelic during the experience, followed by LSD. Depression and anxiety were the most common psychiatric diagnoses, being reported by 73% of ‘SRI –’ subjects and 97% of ‘SRI +’ subjects. Baseline WEMWBS scores (*t*(90) = 1.91, *p* = 0.06) and baseline QIDS-SR-16 scores (*t*(90) = −1.61, *p* = 0.11) were not significantly different between ‘SRI –’ and ‘SRI +’ subjects, indicating that the two groups were comparable at baseline. Detailed demographics are shown in Table 1.

**Table 1.** Demographic information collected at baseline for the survey participants.

Total	SRI − (*N* = 98)	SRI + (*N* = 63)
Age	32.6±11.5	36.7±14.2
Gender		
Female	41(41.8%)	32 (50.8%)
Male	54 (55.1%)	31 (49.2%)
Other	3 (3.1%)	0 (0%)
Nationality		
United States	30 (30.6%)	28 (44.5%)
United Kingdom	29 (29.6%)	16 (25.4%)
Denmark	8 (8.2%)	0 (0%)
Canada	6 (6.1%)	4 (6.3%)
Germany	6 (6.1%)	0 (0%)
Netherlands	6 (6.1%)	2 (3.2%)
Other (14 countries)	13 (13.3%)	13 (20.6%)
Employment status		
Full-time job	41 (41.8%)	26 (41.3%)
Student	27 (27.5%)	13 (20.6%)
Part-time job	18 (18.4%)	11 (17.5%)
Unemployed	9 (9.2%)	6 (9.5%)
Retired	3 (3.1%)	7 (11.1%)
Psychiatric history		
MDD	29 (29.6%)	32 (50.8%)
Anxiety	42 (42.9%)	30 (47.6%)
Eating disorder	15 (15.3%)	1 (1.6%)
OCD	13 (13.3%)	2 (3.2%)
ADHD	5 (5.1%)	15 (23.8%)
Substance abuse disorder	16 (16.3%)	3 (4.8%)
Personality disorder	3 (3.1%)	2 (3.2%)
Bipolar	10 (10.2%)	11 (17.5%)
HPPD	4 (4.1%)	3 (4.8%)
Alcohol dependence	3 (3.1%)	1 (1.6%)
Schizophrenia	0 (0%)	1 (1.6%)
Psychotic disorder	0 (0%)	1 (1.6%)
Previous psychedelic drug use		
Never	21 (21.4%)	16 (25.4%)
Only once	5 (5.1%)	8 (12.7%)
2–5 times	19 (19.5%)	15 (23.8%)
6–10 times	15 (15.3%)	8 (12.7%)
11–20 times	13 (13.3%)	9 (14.3%)
21–50 times	17 (17.3%)	7 (11.1%)
51–100 times	6 (6.1%)	0 (0%)
More than 100 times	2 (2.0%)	0 (0%)
Substance used		
Psilocybin	49 (50%)	45 (71.4%)
LSD/1P-LSD	34 (34.7%)	13 (20.6%)
Ayahuasca	14 (14.2%)	2 (3.2%)
DMT/5-MeO-DMT	1 (1.1%)	3 (4.8%)
Well-being		
WEMWBS baseline	44.1 ± 10.5	39.7 ± 10.3
Depressive symptoms		
QIDS-SR-16 baseline	8.12 ± 4.4	9.97 ± 5.7

The values demonstrated in the table are mean age (±SD) and absolute frequencies. The numbers in brackets are the percentage values.

### Selection of potential confounding variables

Independent samples *t*-tests were used to identify potentially confounding variables that differed between ‘SRI +’ and ‘SRI −’ subjects. Results showed that ‘SRI +’ subjects were significantly older than ‘SRI −’ subjects (*t*(129) = −1.99, *p* = 0.048) and ‘SRI +’ subjects reported more therapeutic intention than ‘SRI −’ subjects (*t*(129) = −3.41, *p* = 0.001) group. On the other hand, ‘SRI −’ subjects reported significantly more frequent psychedelics use (*t*(129) = −0.32, *p* = 0.020), curiosity about the experience (*t*(129) = 2.15, *p* = 0.032), connection with nature (*t*(129) = 2.50, *p* = 0.015), listening to music during the experience (*t*(129) = 2.60, *p* = 0.010) and the presence of emotional support during the experience (*t*(129) = 2.340, *p* = 0.021) than ‘SRI +’ subjects. None of the other variables related to participant demographics and set and setting, including dose of the psychedelic were statistically different across the two groups; therefore, they were not included in further analyses. To test multicollinearity among identified covariates, a separate linear regression was constructed and VIF values were checked. None of the VIF values were higher than 5; therefore, all the possible covariates were included in the MANCOVA analysis. Full analyses on confounding variables and VIF estimates are shown in Supplemental Tables 1 and 2.

### Primary outcome measures

#### Effects of SRI use on the subjective psychedelic experience

**Figure 2.**
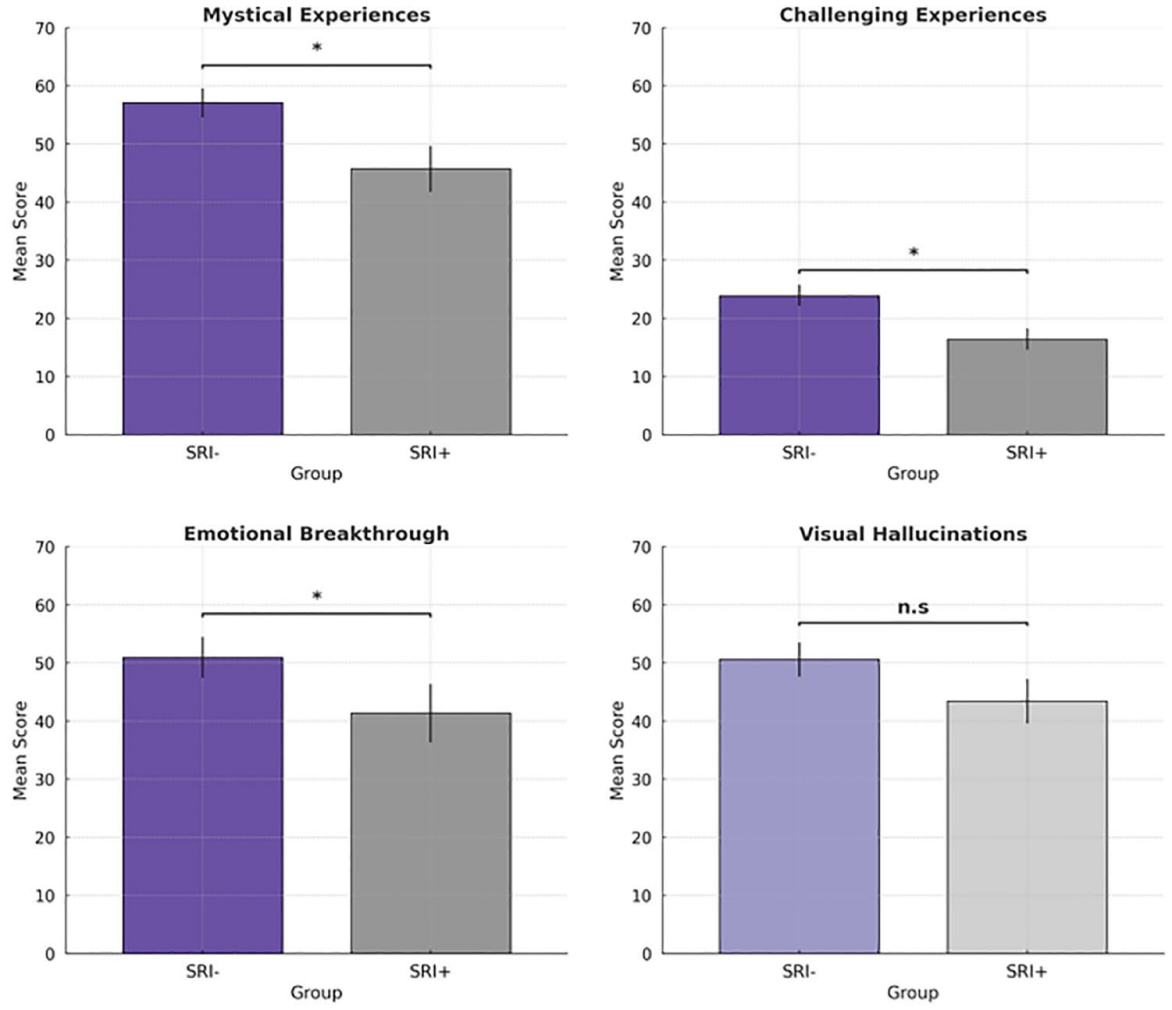
Results for MANCOVA conducted for participants who are SRI-naive (*n* = 84) and currently on SSRI/SNRI (*n* = 47) taking classic psychedelics during their experience. Participants treated with SRIs at baseline had significantly lower scores in the MEQ, CEQ and EBI. Drug-induced visual alterations (ASC-Vis) did not differ between the two groups. Error bars (I) indicate the standard error and the asterisk (*) indicates the significant difference between SRI-naive and SRI users with a *p* < 0.05.

Out of 161 eligible subjects, only 131 of them answered the acute subjective questionnaires 1 day after the experience. In all, 84 subjects classified as ‘SRI –’ and 47 subjects classified as ‘SRI +’. An initial Box test to check for the assumption of homogeneity of variances and covariances in the MANOVA revealed that the assumptions were not violated (Box’s *M* = 11.5, *p* = 0.35). While controlling for confounding variables, MANCOVA results showed a significant Time × Condition interaction, suggesting a difference in intensity scores assessing different facets of the psychedelic experience between ‘SRI +’ and ‘SRI–’ subjects (*p* = 0.016, $\eta_{p}^{2}$ = 0.09) (Table 2). Follow-up analyses showed that ‘SRI +’ subjects had significantly lower scores on the MEQ (F_(1,124)_ = 3.997, *p* = 0.048, $\eta_{p}^{2}$ = 0.03), CEQ (*F*(1, 124) = 10.618, *p* = 0.001, $\eta_{p}^{2}$ = 0.08) and EBI (*F*(1, 124) = 5.772, *p* = 0.018, $\eta_{p}^{2}$ = 0.04) (Figure 2; Supplemental Tables 3 and 4). However, no between-group significant differences were found in ASC-Vis scores (*F*(1, 124) = 1.666, *p* = 0.199, $\eta_{p}^{2}$ = 0.01).

**Table 2.** MANCOVA results.

Effect	Value	*F*	Hypothesis df	Error df	Significance	Partial effect size ( $\eta_{p}^{2}$ )
Antidepressant medication history (SSRI/SNRI)	0.09	3.200	4	119	0.016*	0.097
Age	0.02	0.677	4	119	0.609	0.022
Therapeutic	0.00	3.055	4	119	0.020*	0.093
Previous psychedelic drug use	0.03	0.966	4	119	0.429	0.031
Emotionally supportive individuals influence	0.08	2.489	4	1119	0.047*	0.077
Listening to music	0.07	2.244	4	119	0.068	0.070
Curiosity	0.03	0.960	4	119	0.432	0.031
Connection with nature	0.14	4.994	4	119	0.001*	0.144

* *p* < 0.05.

### Secondary results

#### Changes in well-being and depressive symptoms

Out of 161 eligible subjects, only 92 of them answered both baseline and 4-week questionnaires. In all, 59 of the subjects self-reported to be ‘SRI −’ and 33 of the subjects reported to ‘SRI +’.

**Figure 3.**
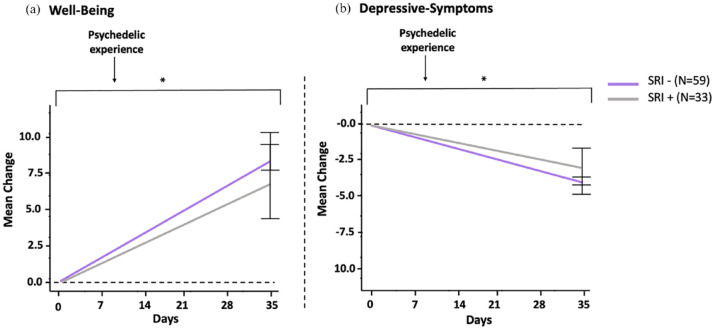
(a, b) Changes in well-being and depression mean scores from baseline to 4-week post-experience. Mean change scores of WEMWBS and QIDS-SR-16 for SRI-naive (*n* = 59) and SRI-users (*n* = 33) between baseline and 4-week follow-up. The results indicate that improvements in well-being and depressive symptoms after a psychedelic experience in the two study groups were comparable. Higher WEMWBS scores depict greater mental well-being, and higher QIDS-SR-16 scores depict greater depression severity. Error bars (I) indicate the standard errors. *p < 0.05.

Table 3(a) presents the results of the linear mixed model predicting WEMWBS scores based on ‘SRI +’ versus ‘SRI –’. After controlling for confounding variables (Supplemental Tables 1 and 2), a significant main effect of time on WEMWBS scores was found (*p* < 0.001). However, the Time × Condition interaction was found to not be significant (*p* = 0.47). This indicates that there were no significant differences in improvements in well-being before and after the psychedelic experience between ‘SRI −’ and ‘SRI +’ subjects (Figure 3).

**Table 3.** Results of the linear mixed model with WEMWBS as the outcome variable, and the following predictor variables: SRI users, significant covariates and time (4-week follow-up).

(a) Well-being (WEMWBS)				
Parameter	Estimate (SE)	*t*	95% CI	*p*
Condition (SRI +)^^^	−3.45 (2.42)	−1.42	−3.09, 2.76	0.16
Connection with nature	−0.68 (0.62)	−1.42	−1.85, 0.47	0.27
Curiosity	0.50 (0.70)	0.71	−0.80, 1.81	0.27
Age	0.07 (0.07)	0.94	−0.07, 0.21	0.254
Previous psychedelic use	−0.05 (0.51)	−0.09	−1.02, 0.91	0.92
Therapeutic intention	−0.02 (0.77)	−0.03	−1.46, 1.41	0.97
Supportive individuals	−2.31 (3.73)	−0.62	−9.29, 4.66	0.53
Listening to music	−0.03 (0.01)	−0.01	−4.09, 4.02	0.98
Time				
Week 4^^^	**7.05 (1.23)**	**7.87**	**4.64, 9.46**	**<0.001** **
Time × Condition				
Week 4^^^	1.47 (2.06)	0.71	−2.56, 5.52	0.47
(b) Depressive symptoms (QIDS-SR-16)				
Condition (SRI +)^^^	1.52 (1.04)	1.46	−0.46, 3.51	0.14
Nature	0.67 (0.26)	2.56	0.17, 1.16	0.01*
Age	−0.02 (0.01)	−0.89	−0.04, 0.01	0.37
Previous psychedelic use	0.08 (0.20)	0.41	−0.32, 0.49	0.68
Therapeutic intention	0.24 (0.32)	0.76	−0.36, 0.85	0.45
Listening to music	−0.68 (0.89)	−0.76	−2.39, 1.02	0.44
Time				
Week 4^^^	**−3.55 (0.62)**	**4.05**	**−4.67**, **−2.33**	**<0.001** **
Time × Condition				
Week 4^^^	−0.88 (1.04)	−0.81	−2.92, 1.15	0.39

While a significant effect of time on WEMWBS scores was found, no differences were found between study groups, as shown by the non-significant Time × SRI users interaction, indicating that improvements in well-being after the psychedelic experience in the two study groups were comparable.

^ Presented for reference condition (SRI users).

* *p* < 0.05. **p < 0.001.

Table 3(b) presents the results of the linear mixed model for QIDS-SR-16 including ‘SRI −’ and ‘SRI +’ subjects. After controlling for significant covariates, a significant main effect of time on depression scores was found (*p* < 0.001). However, the Time × Condition interaction was found to not be significant (*p* = 0.39). This indicates that there were no significant differences in improvements in depressive symptoms before and after the psychedelic experience between ‘SRI −’ and ‘SRI +’ subjects (Figure 3).

## Discussion

The present study examined potential differences in the quality of acute subjective psychedelic experiences between individuals self-reporting psychiatric diagnoses who have never been treated with SRI medications (defined as ‘SRI −’), and those currently undergoing treatment with SRIs (defined as ‘SRI +’). ‘SRI −’ subjects showed significantly more intense acute subjective psychedelic experiences compared to ‘SRI +’ subjects. Specifically, compared to subjects who were using SRIs at baseline, ‘SRI −’ had significantly more intense mystical experiences (18.2% more intense), challenging experiences (50.9% more intense) and emotional breakthroughs (31.9% more intense), with small to moderate effect sizes. No significant differences between the groups were found for drug-induced visual alterations (Figure 2). The study further investigated the before–after changes in well-being and depressive symptoms in these two groups. However, we did not find significant differences between ‘SRI −’ and ‘SRI +’ subjects for improvements in well-being and depressive symptoms after the psychedelic experience; the two groups showed comparable improvements (Figure 3).

These results are consonant with early reports suggesting that chronic treatment with SRIs might reduce the subjective effects of psychedelics (Bonson et al., 1996; Strassman, 1992) and with a recent survey study showing that concurrent use of SSRIs/SNRIs weakened psilocybin’s effects in about half of the study subjects (Gukasyan et al., 2023). However, these results are partially at odds with a recent randomised controlled trial (Becker et al., 2022) indicating that pre-treatment with the SSRI escitalopram had no relevant impact on positive effects of psilocybin, but significantly reduced ratings of any drug effect and bad drug effects (conceptually similar to the reductions in challenging experiences found in the present study). Pre-treatment with escitalopram also reduced the physiological effects of psilocybin (heart rate and pupil size). While our results originate from naturalistic psychedelic use in uncontrolled settings, the sample of (Becker et al., 2022) was small (*N* = 23) and only consisted of healthy subjects who were treated with escitalopram for just 2 weeks, possibly not accounting for long-term changes in brain chemistry and receptor expression. Furthermore, the study only tested escitalopram, limiting generalisability to other SRIs like SNRIs.

There are a few possible explanations for the present results which we will discuss herein. Previous research showed that chronic administration of SSRIs and SNRIs induces down-regulation and desensitisation of several 5-HT receptors (Fritze et al., 2017; Gray & Roth, 2001). Desensitisation refers to the process where 5-HT receptors, due to continuous exposure to these medications, may become less responsive or ‘desensitised’ to 5-HT. This is a rapidly reversible process, meaning the receptors can quickly regain their original sensitivity once the administration of the medication ceases. Down-regulation, conversely, signifies a reduction in the total number of 5-HT receptors present on the cell surface. This phenomenon occurs due to continuous exposure to SSRIs and SNRIs, leading to fewer receptors available for binding. Recovery from down-regulation is considerably slower because it requires the synthesis of new receptors (Fritze et al., 2017; Gray & Roth, 2001). Both pre-clinical (Klimek et al., 1994; Kubota et al., 1989; Wamsley et al., 1987) and clinical (Meyer et al., 2001) research suggests that chronic use of SRIs might induce down-regulation and desensitisation of 5-HT_2A_ receptors. However, this has not been found consistently (Moresco et al., 2000; Zanardi et al., 2001). In addition, pre-clinical (González-Maeso et al., 2007) and clinical studies (Kometer et al., 2013), including positron emission tomography imaging studies (Madsen et al., 2019), suggest that psychedelics exert their acute emotional and visual alterations by stimulating 5-HT_2A_ receptors. Specifically, the intensity of acute psychedelic effects has been demonstrated to be directly associated with 5-HT_2A_ receptor occupancy in the human brain (Madsen et al., 2019). Thus, it is plausible that the chronic use of SRI medications may impair the intensity of the acute psychedelic experience due to 5-HT_2A_ receptor down-regulation and desensitisation. However, our findings indicate that the reduced intensity of the acute subjective psychedelic experience in SRI users is specific to the emotional components of the experience (MEQ, EBI, CEQ), while drug-induced visual alterations did not significantly differ in the two groups. Therefore, it is unlikely that a widespread down-regulation of 5-HT_2A_ receptors can fully account for the present results.

An alternative explanation for our findings may be related to changes in emotional responsivity following SRI treatment. A commonly reported side effect of SRIs is indeed emotional blunting, which is defined as a reduced ability to experience both positive and negative emotions (Opbroek et al., 2002; McCabe et al., 2010). Therefore, we speculate that SRI-induced emotional blunting specifically reduces the intensity of both positive and challenging emotional components of the acute psychedelic experience while leaving the drug-induced visual effects unchanged. Although one could argue that reducing the intensity of challenging experiences induced by psychedelics may be beneficial for patients, previous research has suggested that certain aspects of a challenging psychedelic experience may be associated with subsequent improvements in well-being (Barrett et al., 2016; Carbonaro et al., 2016; Gashi et al., 2021).

Despite the significant differences in the intensity of emotional components of the acute subjective experience between the two groups, improvements in depressive symptoms and well-being before and after psychedelic use were comparable. This is consistent with a recent study on treatment-resistant depression that found that psilocybin therapy, given as an adjunctive treatment to SSRI therapy, produced similar decreases in depressive symptoms as when psilocybin therapy was administered to patients not currently on medications (Goodwin et al., 2023). While it is generally believed that higher ratings of subjective psychedelic effects are associated with higher long-term improvements, this relationship has not been consistently found in research (Griffiths et al., 2008; Gukasyan et al., 2022; Sloshower et al., 2023). Furthermore, it is conceivable that the observed reductions in certain facets of the psychedelic experience among ‘SRI +’ subjects were not so intense as to impede the therapeutic effects of psychedelics, leading to equivalent post-experience improvements. This hypothesis is further supported considering that both groups presented an average severity of depressive symptoms that ranged from mild to moderate at baseline, likely not presenting a particularly complex population.

There is growing clinical evidence that psychedelic-assisted therapies might benefit patients suffering from depression, anxiety and PTSD (Nutt et al., 2020), and it is common clinical practice to treat patients diagnosed with these conditions with SRIs. Therefore, it is important to understand if candidates for psychedelic therapy currently being treated with SRIs should come off their medications before being administered a psychedelic compound. While it is common practice to stop taking SRIs at least 2 weeks before the psychedelic experience in recent clinical trials (Carhart-Harris et al., 2016, 2021; Davis et al., 2021; Malcolm & Thomas, 2022), we previously found that discontinuing SRIs before trial start negatively impacted the outcomes, likely due to the emergence of discontinuation symptoms (Erritzoe et al., in press). Additionally, Goodwin et al. (2023) found in an exploratory study that the combination of psilocybin with SSRIs appeared effective and well-tolerated. These findings thus raise the question of whether it might be more prudent to continue subjects on SRIs, possibly at a reduced dose, rather than completely discontinuing them prior to psilocybin-assisted therapy. An alternative approach may entail suggesting patients longer tapering periods with hyperbolic reductions of medication dose (Groot and van Os, 2021; Horowitz and Taylor, 2019) or regimens involving partial tapering focused on dose reduction rather than complete discontinuation. However, this approach also poses challenges, including prolonged treatment gaps prior to psychedelic therapy and might require re-titration of an antidepressant in case of lack of/limited effects of the psychedelic intervention.

## Limitations

The study presented several limitations worth noting. Analyses of survey outcomes were not pre-registered or adjusted for multiplicity from earlier publications, raising potential type I errors. Thus, our results should be viewed as exploratory, warranting further replication. Despite using the Box’s *M* test and finding no significant variance in homogeneity, the unequal sizes of our sample groups, especially with the larger SRI group, could have impacted the findings. Participants reported on their SRI use a week before their psychedelic experience without controlled verification. However, given (Gukasyan et al., 2023) findings on the lingering subjective effects after SRI discontinuation, our results might still hold consistent. A significant data gap existed, as we did not have information on how long the ‘SRI +’ group had been on their medication. While all participants had a mental health disorder, suggesting prolonged medication use, this absence could influence the study’s conclusions. Therefore, future studies should inquire about the time period subjects were on their medication, whether they stopped/paused taking their medication prior to psychedelic drug exposure, and, if stopped, how many days before the psychedelic experience they discontinued their medications. Furthermore, the generalisability of our results is restricted due to the self-reported mild to moderate severity of depressive symptoms. Some participants did not complete every survey stage, affecting our sample size for particular analyses. Lastly, instead of directly assessing psychedelic doses, we depended on subjective reports. This method has inherent issues, such as inaccurate dose estimations. Future research should consider using predefined dose intervals for different drugs in their surveys.

## Conclusion

The present study suggests that individuals currently medicated with SRIs experienced a significantly less intense subjective experience in the domains of mystical-type experiences, challenging experiences and emotional breakthroughs when compared to those who were never treated with SRIs. With regard to long-term changes, both study populations demonstrated comparable improvements in depressive symptoms and well-being following the psychedelic experience. These findings are exploratory in nature and were obtained from non-controlled settings and may reflect subjects’ self-finding of their experience and desire for a positive impact. Future research utilising controlled methodology especially in clinical populations is now needed. This information will help optimise the implementation of psychedelic-assisted therapy in clinical practice.

## Supplemental Material

sj-docx-1-jop-10.1177_02698811231224217 – Supplemental material for Interactions between classic psychedelics and serotonergic antidepressants: Effects on the acute psychedelic subjective experience, well-being and depressive symptoms from a prospective survey study Click here for additional data file. Supplemental material, sj-docx-1-jop-10.1177_02698811231224217 for Interactions between classic psychedelics and serotonergic antidepressants: Effects on the acute psychedelic subjective experience, well-being and depressive symptoms from a prospective survey study by Jessica Barbut Siva, Tommaso Barba, Hannes Kettner, Joanna Kuc, David J Nutt, Robin Carhart-Harris and David Erritzoe in Journal of Psychopharmacology
