# Supplementary material for: Interactions between classic psychedelics and serotonergic antidepressants: Effects on the acute psychedelic subjective experience, well-being and depressive symptoms from a prospective survey study
Source: J Psychopharmacol. 2024 Jan 27;38(2):145–55. doi: 10.1177/02698811231224217 (PMC10863370; doi:10.1177/02698811231224217)
Supplement: sj-docx-1-jop-10.1177_02698811231224217 – Supplemental material for Interactions between classic psychedelics and serotonergic antidepressants: Effects on the acute psychedelic subjective experience, well-being and depressive symptoms from a prospective survey study [file sj-docx-1-jop-10.1177_02698811231224217.docx]

**Supplementary Material**

**Table 1|** Confounding variables between “SRI +” and “SRI –“ subjects.

**SRI - SRI +**

Mean ±SD Mean ±SD t. Sig.

Age 32.65 11.44 36.71 14.22 -1.99 .048*

Gender 0.48 0.56 0.51 0.50 -0.32 .745

Previous psychedelic use 2.81 2.00 2.11 1.69 2.35 .020*

Dose of psychedelic 4.05 1.49 4.32 1.49 -0.76 .446

**Setting** Party 1.40 1.48 1.16 1.40 1.02 .310

Social 1.20 1.40 0.97 1.42 0.99 .332 Spiritual 2.73 1.29 2.79 1.35 -0.27 .786

Religious 0.64 1.19 0.40 0.87 1.34 .181

**Intention** Therapeutic 2.72 1.34 3.39 0.93 -3.41 .001*

Curiosity 2.71 1.33 2.23 1.45 2.15 .032*

Escape from emotions 0.74 1.26 1.15 1.45 -1.84 .066

Connection with nature 2.67 1.50 2.06 1.57 2.50 .015*

**Environmental** Listening to music 0.69 0.46 0.49 0.50 2.60 .010*

Emotional support 0.52 0.50 0.30 0.46 2.78 .006*

Perceived threat 0.05 0.22 0.03 1.17 0.58 .561

Setting, intention, and environmental confounding variable scoring = 0 = strongly disagree, 1 = disagree, 2 = neither agree nor disagree, 3 = agree, 4 = strongly agree

* = p< 0.05, SD = standard deviation

SRI = serotonin reuptake inhibitors

**Table 2|** Multicollinearity and regression diagnostics

|  | **VIF** |
| --- | --- |
| **Age** | 1.109 |
| **Number of previous psychedelic use** | 1.054 |
| **Therapeutic** | 1.042 |
| **Listening to music** | 1.873 |
| **Emotionally supportive individual** | 1.848 |
| **Curiosity** | 1.036 |
| **Connection with nature** | 1.099 |

**Table 3|** Dependent variables in MANCOVA model.

| **Source** | **Dependent Variable** | **df** | **Mean Square** | **F** | **Sig.** | **Partial Eta Squared** |
| --- | --- | --- | --- | --- | --- | --- |
| **SRI medication history** | MEQ% Max Scores | 1 | 2169.303 | 3.997 | 0.048 | 0.032 |
|  | CEQ% Max Scores | 1 | 2480.656 | 10.618 | 0.001 | 0.080 |
|  | EBI**%** Max Scores | 1 | 5748.815 | 5.772 | 0.018 | 0.045 |
|  | ASC% Max Scores | 1 | 1141.112 | 1.666 | 0.199 | 0.013 |
| **Age** | MEQ% Max Scores | 1 | 410.778 | 0.757 | 0.386 | 0.006 |
|  | CEQ% Max Scores | 1 | 74.838 | 0.320 | 0.572 | 0.003 |
|  | EBI**%** Max Scores | 1 | 11.165 | 0.011 | 0.916 | 0.000 |
|  | ASC% Max Scores | 1 | 111.849 | 0.163 | 0.687 | 0.001 |
| **Previous psychedelic drug use** | MEQ% Max Scores | 1 | 590.825 | 1.089 | 0.299 | 0.009 |
|  | CEQ% Max Scores | 1 | 8.197 | 0.035 | 0.852 | 0.000 |
|  | EBI**%** Max Scores | 1 | 63.275 | 0.064 | 0.801 | 0.001 |
|  | ASC% Max Scores | 1 | 131.317 | 0.192 | 0.662 | 0.002 |
| **Therapeutic** | MEQ% Max Scores | 1 | 427.432 | 0.788 | 0.377 | 0.006 |
|  | CEQ% Max Scores | 1 | 156.503 | 0.670 | 0.415 | 0.005 |
|  | EBI**%** Max Scores | 1 | 10379.200 | 10.421 | 0.002 | 0.079 |
|  | ASC% Max Scores | 1 | 960.272 | 1.402 | 0.239 | 0.011 |
| **Curiosity** | MEQ% Max Scores | 1 | 97.450 | 0.180 | 0.672 | 0.001 |
|  | CEQ% Max Scores | 1 | 492.540 | 2.108 | 0.149 | 0.017 |
|  | EBI**%** Max Scores | 1 | 301.159 | 0.302 | 0.583 | 0.002 |
|  | ASC% Max Scores | 1 | 156.004 | 0.228 | 0.634 | 0.002 |
| **Connection with nature** | MEQ% Max Scores | 1 | 9163.370 | 16.886 | 0.000 | 0.122 |
|  | CEQ% Max Scores | 1 | 24.684 | 0.106 | 0.746 | 0.001 |
|  | EBI**%** Max Scores | 1 | 3444.189 | 3.458 | 0.065 | 0.028 |
|  | ASC% Max Scores | 1 | 6701.551 | 9.782 | 0.002 | 0.074 |
| **Emotionally supportive individual influence** | MEQ% Max Scores | 1 | 267.460 | 0.493 | 0.484 | 0.004 |
|  | CEQ% Max Scores | 1 | 443.256 | 1.897 | 0.171 | 0.015 |
|  | EBI**%** Max Scores | 1 | 2023.008 | 2.031 | 0.157 | 0.016 |
|  | ASC% Max Scores | 1 | 1540.060 | 2.248 | 0.136 | 0.018 |
| **Listening to music** | MEQ% Max Scores | 1 | 406.339 | 0.749 | 0.389 | 0.006 |
|  | CEQ% Max Scores | 1 | 659.378 | 2.822 | 0.096 | 0.023 |
|  | EBI**%** Max Scores | 1 | 5781.498 | 5.805 | 0.017 | 0.045 |
|  | ASC% Max Scores | 1 | 32.938 | 0.048 | 0.827 | 0.000 |

MEQ = Mystical Experience Questionnaire, CEQ = Challenging Experience Questionnaire, EBI = Emotional Breakthrough Inventory, ASC-Vis = Visual subscales of the Altered States of Consciousness Questionnaire.

*= p<0.005

**Table 4|** Mean values of acute subjective measures in antidepressant naive and current antidepressant users.

**Dependent Antidepressant Mean Std. Error 95% Confidence Interval Variable Medication History Lower Bound | Upper Bound**

**(SSRI/SNRI)**

MEQ % Max SRI - 56.38 2.65 51.14 61.61

Scores SRI + 46.96 3.64 39.75 54.17

CEQ % Max SRI - 24.83 1.74 21.39 28.27

Scores SRI + 14.76 2.39 10.03 19.49

EBI % Max SRI - 52.01 3.58 45.92 60.11

Scores SRI + 37.68 4.93 27.92 47.45

Visual-ASC SRI - 50.49 2.97 44.60 56.37

% Max Scores SRI + 43.66 4.10 35.56 51.76

MEQ = Mystical Experience Questionnaire, CEQ = Challenging Experience Questionnaire, EBI = Emotional Breakthrough Inventory, ASC-Vis = Visual subscales of the Altered States of Consciousness Questionnaire.
